# Opposing prognostic relevance of junction plakoglobin in distinct prostate cancer patient subsets

**DOI:** 10.1002/1878-0261.12922

**Published:** 2021-02-17

**Authors:** Tanja Spethmann, Lukas Clemens Böckelmann, Vera Labitzky, Ann‐Kristin Ahlers, Jennifer Schröder‐Schwarz, Sarah Bonk, Ronald Simon, Guido Sauter, Hartwig Huland, Robert Kypta, Udo Schumacher, Tobias Lange

**Affiliations:** ^1^ Institute of Anatomy and Experimental Morphology Center for Experimental Medicine University Cancer Center Hamburg University Medical Center Hamburg‐Eppendorf Germany; ^2^ Martini‐Klinik, Prostate Cancer Center University Medical Center Hamburg‐Eppendorf Germany; ^3^ General, Visceral and Thoracic Surgery Department University Medical Center Hamburg‐Eppendorf Germany; ^4^ Institute of Pathology University Medical Center Hamburg‐Eppendorf Germany; ^5^ Department of Surgery and Cancer Imperial College London UK; ^6^ Center for Cooperative Research in Biosciences CIC bioGUNE Derio Spain

**Keywords:** cell adhesion, CHD1, ERG, junction plakoglobin, prostate cancer, WNT signaling

## Abstract

Both oncogenic and tumor suppressor functions have been described for junction plakoglobin (JUP), also known as γ‐catenin. To clarify the role of JUP in prostate cancer, JUP protein expression was immunohistochemically detected in a tissue microarray containing 11 267 individual prostatectomy specimens. Considering all patients, high JUP expression was associated with adverse tumor stage (*P* = 0.0002), high Gleason grade (*P* < 0.0001), and lymph node metastases (*P* = 0.011). These associations were driven mainly by the subset without *TMPRSS2:ERG* fusion, in which high JUP expression was an independent predictor of poor prognosis (multivariate analyses, *P* = 0.0054) and early biochemical recurrence (*P* = 0.0003). High JUP expression was further linked to strong androgen receptor expression (*P* < 0.0001), high cell proliferation, and *PTEN* and *FOXP1* deletion (*P* < 0.0001). In the ERG‐negative subset, high JUP expression was additionally linked to *MAP3K7* (*P* = 0.0007) and *CHD1* deletion (*P* = 0.0021). Contrasting the overall prognostic effect of JUP, low JUP expression indicated poor prognosis in the fraction of *CHD1*‐deleted patients (*P* = 0.039). In this subset, the association of high JUP and high cell proliferation was specifically absent. In conclusion, the controversial biological roles of JUP are reflected by antagonistic prognostic effects in distinct prostate cancer patient subsets.

## Graphical abstract

Junction plakoglobin (JUP) is a component of desmosomes and adherens junctions and reported to play divergent roles in tumorigenesis. JUP expression was analyzed in prostate cancer in a large‐enough patient cohort to reflect the molecular heterogeneity of the disease. Overall, high JUP expression was associated with adverse tumor features, while low JUP expression indicated poor prognosis in *CHD1*‐deleted patients.

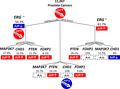

**Abbreviations**
AR: androgen receptor
BCR: biochemical recurrence‐free survival
EMT: epithelial–mesenchymal transition
ERG: ETS transcription factor ERG
GEO: Gene Expression Omnibus database
GEPIA: Gene Expression Profiling Interactive Analysis online tool
GTEx: Genotype‐Tissue Expression database
IHC: immunohistochemistry
JUP: junction plakoglobin/γ‐catenin
PCa: prostate cancer
PSA: prostate‐specific antigen
RP: radical prostatectomy
TCGA: The Cancer Genome Atlas database
TMA: tissue microarray
TMPRSS2: transmembrane protease serine subtype 2

## 1 Introduction

Prostate cancer (PCa) accounts for one‐third of all cancer‐related deaths among men in developed countries [1]. Despite substantial advances in recent years, PCa remains a therapeutic challenge as tumors are biologically heterogeneous and clinical outcomes vary significantly. Localized and locally advanced tumor stages are usually treated by surgery and radiation, but especially men with high‐grade and high‐stage tumors will often experience tumor recurrence after local treatment. Established prognostic factors include preoperative prostate‐specific antigen (PSA) levels, Gleason grade of the tumor, the number of tumor‐containing biopsy cores, tumor stage, and the resection margin. In particular, the Gleason score is one of the most robust predictive markers for the oncological outcome and strongly impacts on clinical decision‐making [2]. The high value of the Gleason grading is compromised, however, by only moderate interobserver reproducibility and intratumoral heterogeneity [3]. Thus, a reliable and clinically applicable molecular marker, operating independently of the Gleason grading and differentially integrating tumor heterogeneity, would be highly desirable.

Junction plakoglobin (JUP), also known as γ‐catenin and a member of the armadillo family of proteins, is a paralog of β‐catenin and the only known constituent of both desmosomes and adherens junctions. In addition to their role in structurally and functionally regulating cell–cell adhesion by linking junctional proteins to the cytoskeleton, catenins are also critically involved in the WNT signaling pathway [4]. Both roles are important in malignant progression as a disruption of adherens junctions and desmosomes can promote cancer cells to dissociate during epithelial‐mesenchymal transition (EMT), and aberrant WNT signaling affects stem cell self‐renewal, cell proliferation, migration, and differentiation [5].

Signaling by secreted WNT proteins is mediated through stabilization and nuclear translocation of the transcription coactivator β‐catenin (canonical pathway). In the nucleus, β‐catenin forms complexes with TCF/LEF and activates WNT target gene expression [4, 6]. (Epi)genetic changes activating β‐catenin‐mediated WNT signaling have been described in many types of cancer, including PCa [7]. However, despite the similarities with β‐catenin, the role of JUP in tumorigenesis remains controversial. Numerous studies found JUP to have properties of a tumor and metastasis suppressor *in vivo*, but overexpression of JUP caused oncogenic activity *in vitro* [8, 9, 10, 11, 12]. While some studies suggested that the oncogenic potential of JUP is more likely associated with increased β‐catenin signaling rather than due to a direct function of JUP, other studies demonstrated oncogenic activity of JUP via pathways that were distinct from that of β‐catenin, but depending on TCF/LEF and c‐Myc function [8, 13]. On the other hand, it has been reported that JUP can inhibit WNT/β‐catenin signaling by several mechanisms and exert part of its tumor suppressor activity through the modulation of apoptosis [4, 14].

These contradictions observed in experimental studies are reflected by clinical studies on the role of JUP in cancer patients. In several tumor entities, the loss of JUP expression resulted in adverse tumor features and was correlated with increased tumor stage, poor patient survival, and increased metastasis [15, 16, 17, 18, 19, 20]. For breast cancer, decreased JUP expression was shown to lower cell–cell contact and thus to increase invasion and cancer cell dissemination *in vivo* [21]. Further, JUP promotes distant metastasis in breast cancer by enhancing the formation of circulating tumor cell clusters [22]. Metastasis‐free survival was significantly lower in breast cancer patients with primary tumors highly expressing JUP [23].

Whether JUP acts as a tumor suppressor or an oncogenic protein may depend on differential expression levels of JUP at different stages of tumorigenesis or within distinct molecular subsets of the same tumor entity. However, the expression level and prognostic significance of JUP in PCa patients are largely unknown [24, 25, 26]. We, therefore, analyzed a tissue microarray composed of 11 267 individual prostatectomy specimens along with paired comprehensive pathological, molecular, and clinical data to characterize the clinical relevance of JUP in PCa in general and in different molecular subsets.

## 2 Materials and Methods

### 2.1 Patients and tissue microarray

In this study, the Hamburg PCa prognosis tissue microarray (TMA) was used, which was expanded from earlier versions [27, 28] by adding further patient samples. Radical prostatectomy (RP) specimens were available from 13 454 patients undergoing surgery between 1992 and 2015 at the Department of Urology and the Martini‐Klinik at the University Medical Center Hamburg‐Eppendorf. All prostatectomy specimens were analyzed according to a standard procedure, including a complete embedding of the entire prostate for histological analysis [29].

The follow‐up data included tumor stage, (quantitative) Gleason grade, nodal status, surgical margin status, and PSA values. In all patients, PSA values were measured quarterly in the first year following surgery, followed by biannual measurements in the second and annual measurements after the third postoperative year. Time to PSA recurrence (biochemical recurrence (BCR)‐free survival) was defined as the time interval between RP and the first occurrence of a postoperative PSA of at least 0.2 ng·mL^−1^ and rising after that. Patients without evidence of tumor recurrence were censored at the time of the last follow‐up.

The molecular database linked to this TMA contained results on ERG expression (IHC) [30], *ERG* break‐apart FISH analysis [31], deletion status of 5q21 (*CHD1*) [32], 6q15 (*MAP3K7*) [33], 10q23 (*PTEN*) [34], 3p13 (*FOXP1*) [35], Ki67‐labeling index data [36], and androgen receptor (AR) expression [30].

TMAs were produced as previously described [37]: Tissue cylinders with a diameter of 0.6 mm were punched randomly from representative tumor or normal areas of each tissue block and brought into a recipient paraffin block. All tumor samples were obtained from the archives of the Institute of Pathology of the University Medical Center Hamburg‐Eppendorf. The use of archived diagnostic left‐over tissues for the manufacturing of TMAs and their analysis for research purposes has been approved by local laws (HmbKHG, §12,1) and by the local ethics committee (Ethics commission Hamburg, WF‐049/09). All work has been carried out in compliance with the Helsinki Declaration.

### 2.2 Immunohistochemistry

Freshly cut sections from all blocks of the Hamburg prostate cancer prognosis TMA were all immunostained as one batch on one day and in one experiment. Slides were deparaffinized and exposed to heat‐induced antigen retrieval for 5 min at 125 °C in pH 6 Target Retrieval Solution (#S1699, Dako, Carpinteria, CA, USA). Primary antibody specific for JUP (rabbit polyclonal antibody, dilution 1 : 50, #HPA032047, Sigma‐Aldrich, Taufkirchen, Germany) was applied at room temperature for 60 min. For isotype control, rabbit IgG antibody (#ab37415, Abcam, Berlin, Germany; diluted 1 : 1000) was applied. After three washes with Tris‐buffered saline, a biotinylated swine anti‐rabbit immunoglobulin (Dako) was incubated on the sections for 30 min at room temperature. After three washing steps with Tris‐buffered saline, an alkaline phosphatase/streptavidin complex (ABC‐AP staining kit, VECTASTAIN, #AK‐5000) was used. Alkaline phosphatase reactivity was then visualized using the Permanent AP‐Red‐kit from ZYTOMED Systems (#ZUC001‐125) according to the manufacturer’s instructions. Finally, sections were counterstained with Mayer’s hemalaun solution.

Junction plakoglobin staining was observed at the cell membranes, and the staining intensity was assessed on a four‐step scale from negative, weak, moderate to strong expression. For correlation with clinic‐pathologic data, the staining intensities were dichotomized (negative to weak = ‘low’, moderate to strong = ‘high’) or trichotomized (negative to weak = ‘low’, moderate, and strong; Tables S2‐4 and Fig. S2). No immunostaining was seen on tissue samples incubated with the isotype control antibody verifying the specificity of the primary anti‐JUP antibody.

### 2.3 Analysis of publicly available gene expression data sets

Gene Expression Profiling Interactive Analysis 2 (GEPIA2) online tool (http://gepia2.cancer‐pku.cn) was used to further verify the expression and correlation of genes. Figure 2A was generated using a log_2_FC cutoff of 0.5 and a *P‐*value cutoff of 0.01. Statistical significance was tested by one‐way ANOVA using disease state (Tumor or Normal) as variable for calculating differential expression. GEO2R online tool (http://www.ncbi.nlm.nih.gov/geo/geo2r) was used to compare JUP mRNA expression in normal prostate tissue, localized primary PCa tissue, and PCa metastases in GEO data sets GDS1439 [38] and GDS2545 [39, 40] (Fig. 2B and C). Pearson correlation analysis of gene expression was carried out using the prostate cancer data set from The Cancer Genome Atlas (TCGA) and using normal prostate samples data sets from TCGA and Genotype‐Tissue Expression (GTEx) portal (Fig. S3). cBioPortal (http://cbioportal.org) was used to compare mRNA expression of WNT target genes *AXIN2, NKD1, LEF1, and MYC*, stratifying patients for JUP mRNA expression and ERG fusion status (Fig. S4). Further, cBioPortal was used to compare JUP mRNA expression alone (Fig. S5) or together with α‐ or β‐catenin mRNA expression and disease‐free status in the TCGA prostate cancer data set (Fig. S6).

### 2.4 Statistics

Statistics were performed with jmp ^®^ 11 software (SAS Institute Inc., Cary, NC, USA). Contingency tables and the chi‐square test (likelihood) were performed to search for associations between molecular parameters and tumor phenotype. Survival curves were calculated according to Kaplan–Meier. The log‐rank test was applied to detect significant differences between groups. Cox proportional hazards regression analysis was performed to test the statistical independence and significance between pathological, molecular, and clinical variables.

## 3 Results

### 3.1 JUP expression in normal and cancerous prostate tissues

**Fig. 1:**
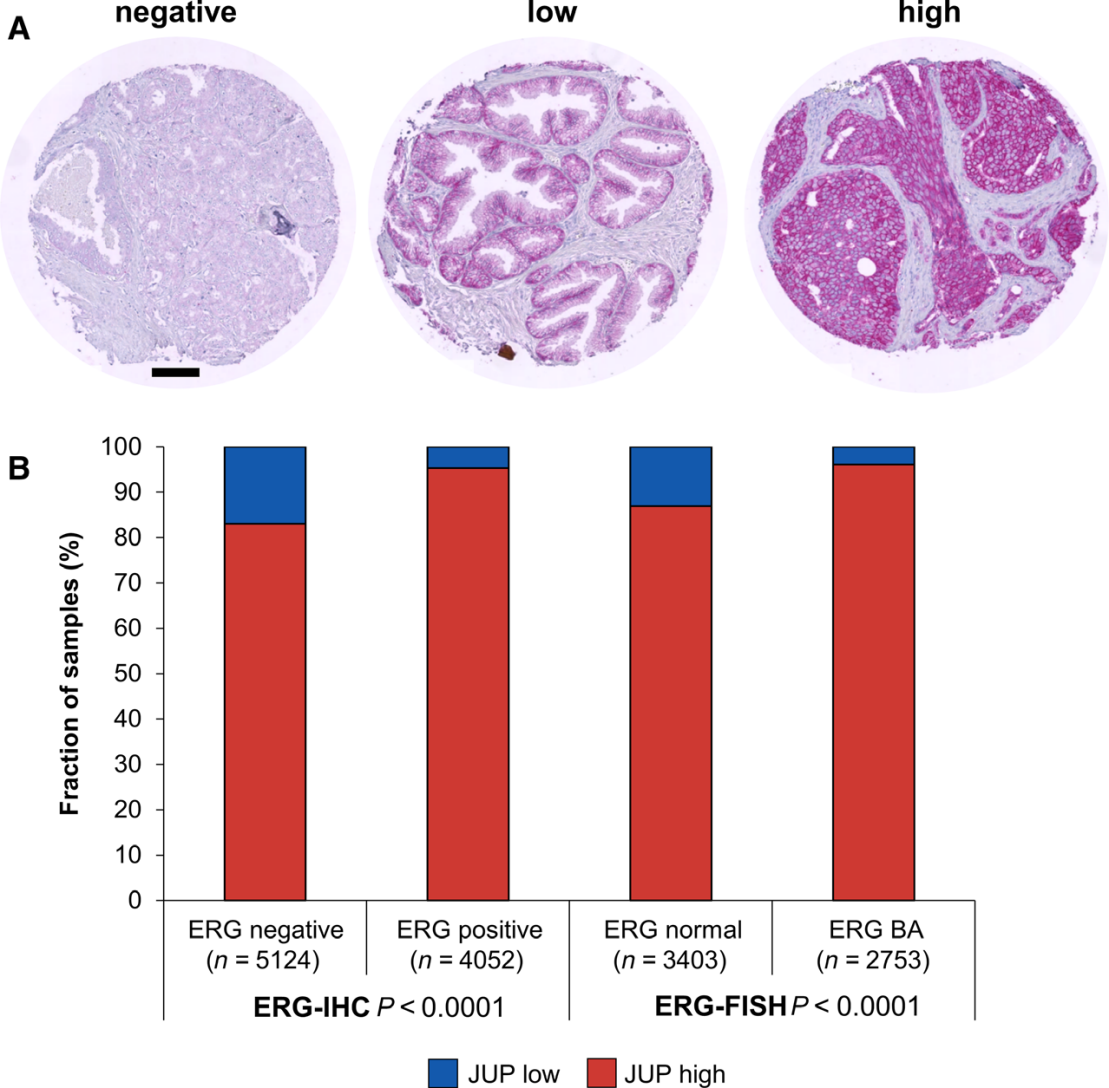
JUP immunostaining in PCa tissue and association between JUP expression and ERG status in all PCa cases. (A) Representative images of JUP immunostaining in cancerous prostate tissue: negative JUP expression (left), low JUP expression (middle), and high JUP expression (right). Scale bar = 100 µm. (B) ERG status was either determined by immunohistochemistry (ERG‐IHC) or by break‐apart (BA) FISH analysis (ERG‐FISH). For association analysis, contingency tables were used and the chi‐square test was performed to test for statistical significance

**Fig. 2:**
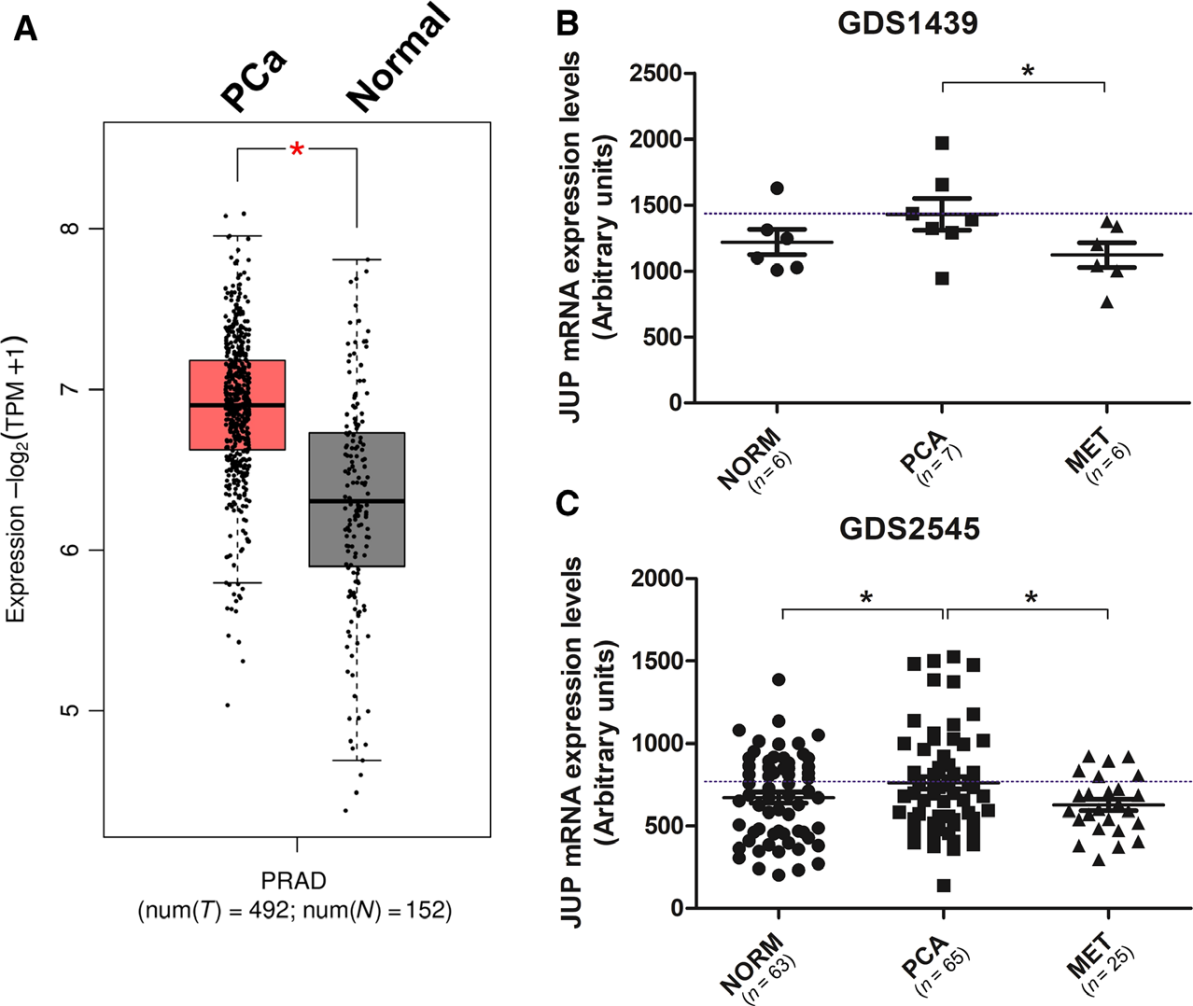
JUP gene expression analysis in publicly available data sets. (A) Gene Expression Profiling Interactive Analysis 2 (GEPIA2) tool was used to compare JUP mRNA expression in PCa (TCGA data) versus normal prostate (TCGA/GTEx combined data). Log_2_FC cutoff = 0.5, *P*‐value cutoff = 0.01. To test for statistical significance, one‐way ANOVA using disease state as variable for calculating differential expression was used. (B and C) Analysis of JUP mRNA expression in normal prostate tissue (NORM), localized primary PCa tissue (PCA), and PCa metastasis tissue (MET) in GEO data sets GDS1439 (B) and GDS2545 (C) using GEO2R (*P < 0.05, unpaired Student’s t‐test)

About 11 267 of 13 454 tumor samples were interpretable in our TMA analysis. Noninformative cases (*n* = 2187; 16.2%) were due to lack of tissue samples, absence of unequivocal cancer tissue in the respective TMA spot, or lack of outcome data or adjusting variables. In normal prostate tissue (*n* = 163), negative to weak (‘low’) JUP expression was detected in 19.6% of all samples and moderate to strong (‘high’) JUP expression was detected in 80.4% of all samples. In PCa tissues, low JUP expression was found in 12.4% of all cases, while high JUP expression was found in 87.6% of all cases (Table 1). Representative IHC images of negative, moderate, and strong JUP expression are shown in Fig. 1A and Fig. S1. These findings were corroborated by Gene Expression Profiling Interactive Analysis (GEPIA) comparing *JUP* gene expression in PCa tissue (TCGA) with normal prostate tissue (TCGA/GTEx), indicating a trend toward increased *JUP* expression in PCa (Fig. 2A). These findings were also validated in GEO data sets GDS1439 and GDS2545, further demonstrating a decrease in *JUP* expression in metastatic PCa lesions compared with samples from localized primary PCa tumors (Fig. 2B and C).

**Table 1:** Associations between JUP immunostaining results and PCa phenotype in all cases analyzed

Parameters	*n* evaluable	Low (0/1) %	High (2/3) %	*P‐*value
All cancers	11 267	12.4	87.6	
*Tumor stage*				
pT2	7285	13.1	86.9	0.0002
pT3a	2486	12.1	87.9	
pT3b‐pT4	1455	9.3	90.7	
*Gleason grade*				
≤3 + 3	2388	15.8	84.2	<0.0001
3 + 4	6066	12.5	87.5	
3 + 4 Tert.5	434	8.5	91.5	
4 + 3	1114	11.0	89.0	
4 + 3 Tert.5	690	8.4	91.6	
≥4 + 4	566	8.1	91.9	
*Quantitative Gleason grade*				
≤3 + 3	2388	15.8	84.2	<0.0001
3 + 4 ≤5%	1627	13.3	86.7	
3 + 4 6–10%	1583	12.4	87.6	
3 + 4 11–20%	1305	12.0	88.0	
3 + 4 21–30%	691	10.6	89.4	
3 + 4 31–49%	575	11.5	88.5	
3 + 4 Tert.5	434	8.5	91.5	
4 + 3 50–60%	489	12.1	87.9	
4 + 3 Tert.5	690	8.4	91.6	
4 + 3 61–100%	508	8.5	91.5	
≥4 + 4	505	7.7	92.3	
*Lymph node metastasis*				
N0	6500	12.9	87.1	0.0113
N+	692	9.7	90.3	
*Preoperative PSA level (ng*·*ml* ^−1^ *)*				
<4	1410	8.9	91.1	<0.0001
4–10	6754	12.2	87.8	
10–20	2245	14.7	85.3	
>20	783	13.2	86.8	
*Surgical margin*				
Negative	8989	12.5	87.5	0.2795
Positive	2237	11.7	88.3	
*Age at time of surgery*				
<50	293	9.6	90.4	0.0831
50–60	2815	11.6	88.4	
60–70	6488	12.5	87.5	
>70	1627	13.7	86.3	

### 3.2 High JUP expression is associated with a more aggressive tumor phenotype

Considering all patients, high JUP expression was associated with adverse tumor features compared to low JUP expression (summarized in Table 1), including advanced tumor stage (*P* = 0.002), high Gleason grade (both conventional and quantitative) (*P* < 0.0001), and presence of lymph node metastases (*P* = 0.011). There was no association between JUP expression and positive surgical margin (*P* = 0.28). Because of the relatively high percentage of patients with low JUP expression in the absence of *ERG* rearrangement (see 3.3), the correlation analysis of JUP and tumor features was repeated in ERG‐negative and ERG‐positive patient subsets. This analysis demonstrated that the association of high JUP expression with adverse tumor features was driven by the subset of ERG‐negative cases (Tables 2 and 3).

**Table 2:** Associations between JUP immunostaining results and PCa phenotype in the *TMPRSS2: ERG* fusion‐negative subset

Parameters	*n* evaluable	Low (0/1) %	High (2/3) %	*P‐*value
All cancers	5124	16.9	83.1	
*Tumor stage*				
pT2	3431	17.7	82.3	0.0017
pT3a	1024	17.2	82.8	
pT3b‐pT4	655	12.2	87.8	
*Gleason grade*				
≤3 + 3	1021	23.6	76.4	<0.0001
3 + 4	2692	17.1	82.9	
3 + 4 Tert.5	227	8.8	91.2	
4 + 3	550	15.5	84.5	
4 + 3 Tert.5	321	9.7	90.3	
≥4 + 4	309	9.7	90.3	
*Quantitative Gleason grade*				
≤3 + 3	1021	23.6	76.4	<0.0001
3 + 4 ≤5%	712	18.3	81.7	
3 + 4 6–10%	707	17.7	82.3	
3 + 4 11–20%	601	15.1	84.9	
3 + 4 21–30%	304	16.8	83.2	
3 + 4 31–49%	267	15.4	84.6	
3 + 4 Tert.5	227	8.8	91.2	
4 + 3 50–60%	234	18.4	81.6	
4 + 3 Tert.5	321	9.7	90.3	
4 + 3 61–100%	262	11.1	88.9	
≥4 + 4	280	8.9	91.1	
*Lymph node metastasis*				
N0	2992	16.7	83.3	0.0206
N+	298	11.7	88.3	
*Preoperative PSA level (ng·ml^−1^)*				
<4	547	13.5	86.5	0.1054
4–10	3018	17.0	83.0	
10–20	1125	18.2	81.8	
>20	407	16.5	83.5	
*Surgical margin*				
Negative	4093	17.1	82.9	0.4475
Positive	1018	16.1	83.9	
*Age at time of surgery*				
<50	104	11.5	88.5	0.4780
50–60	1152	17.2	82.8	
60–70	3059	16.9	83.1	
>70	794	17.3	82.7	

**Table 3:** Associations between JUP immunostaining results and PCa phenotype in the *TMPRSS2: ERG* fusion‐positive subset

Parameters	*n* evaluable	Low (0/1) %	High (2/3) %	*P‐*value
All cancers	4052	4.7	95.3	
*Tumor stage*				
pT2	2419	4.4	95.6	0.4634
pT3a	1072	5.3	94.7	
pT3b‐pT4	544	4.2	95.8	
*Gleason grade*				
≤3 + 3	843	6.6	93.4	0.0291
3 + 4	2321	4.4	95.6	
3 + 4 Tert.5	123	4.1	95.9	
4 + 3	394	4.1	95.9	
4 + 3 Tert.5	220	1.8	98.2	
≥4 + 4	148	4.7	95.3	
*Quantitative Gleason grade*				
≤3 + 3	843	6.6	93.4	0.0334
3 + 4 ≤5%	585	3.8	96.2	
3 + 4 6–10%	612	3.9	96.1	
3 + 4 11–20%	501	4.2	95.8	
3 + 4 21–30%	293	3.4	96.6	
3 + 4 31–49%	208	6.7	93.3	
3 + 4 Tert.5	123	4.1	95.9	
4 + 3 50–60%	177	4.0	96.0	
4 + 3 Tert.5	220	1.8	98.2	
4 + 3 61–100%	174	2.3	97.7	
≥4 + 4	123	4.1	95.9	
*Lymph node metastasis*				
N0	2316	5.0	95.0	0.3508
N+	251	6.4	93.6	
*Preoperative PSA level (ng·ml^−1^)*				
<4	553	3.1	96.9	0.0555
4–10	2494	4.4	95.6	
10–20	722	5.7	94.3	
>20	253	6.7	93.3	
*Surgical margin*				
Negative	3180	4.8	95.2	0.3937
Positive	854	4.1	95.9	
*Age at time of surgery*				
<50	149	7.4	92.6	0.4351
50–60	1149	4.9	95.1	
60–70	2264	4.4	95.6	
>70	471	4.2	95.8	

### 3.3 Low JUP expression is associated with the absence of TMPRSS2:ERG fusion

Data on the *TMPRSS2:ERG* fusion status obtained by FISH were available from 6156 and by IHC from 9176 tumors with interpretable JUP immunostaining. Associations of the presence or absence of the *TMPRSS2:ERG* fusion with clinicopathologic characteristics in the study cohort are provided in Table S1. The absence of *TMPRSS2:ERG* fusion was associated with higher Gleason grade, higher preoperative PSA value, and higher age. Low JUP expression was strongly linked to the absence of *TMPRSS2:ERG* rearrangement or ERG overexpression (determined by FISH or IHC, respectively; Fig. 1B). For instance, the fraction of patients with low JUP expression increased from 4.7 % in 4052 ERG‐positive cases to 16.9% in 5124 ERG‐negative cases (*P* < 0.0001; Fig. 1B, *ERG* status determined by IHC). Analysis of publicly available data sets from The Cancer Genome Atlas (TCGA) found *JUP* and *ERG* gene expression correlated specifically in PCa, but not in benign/normal prostate gland (Fig. S3).

### 3.4 Association of JUP expression with common deletions in PCa

**Fig. 3:**
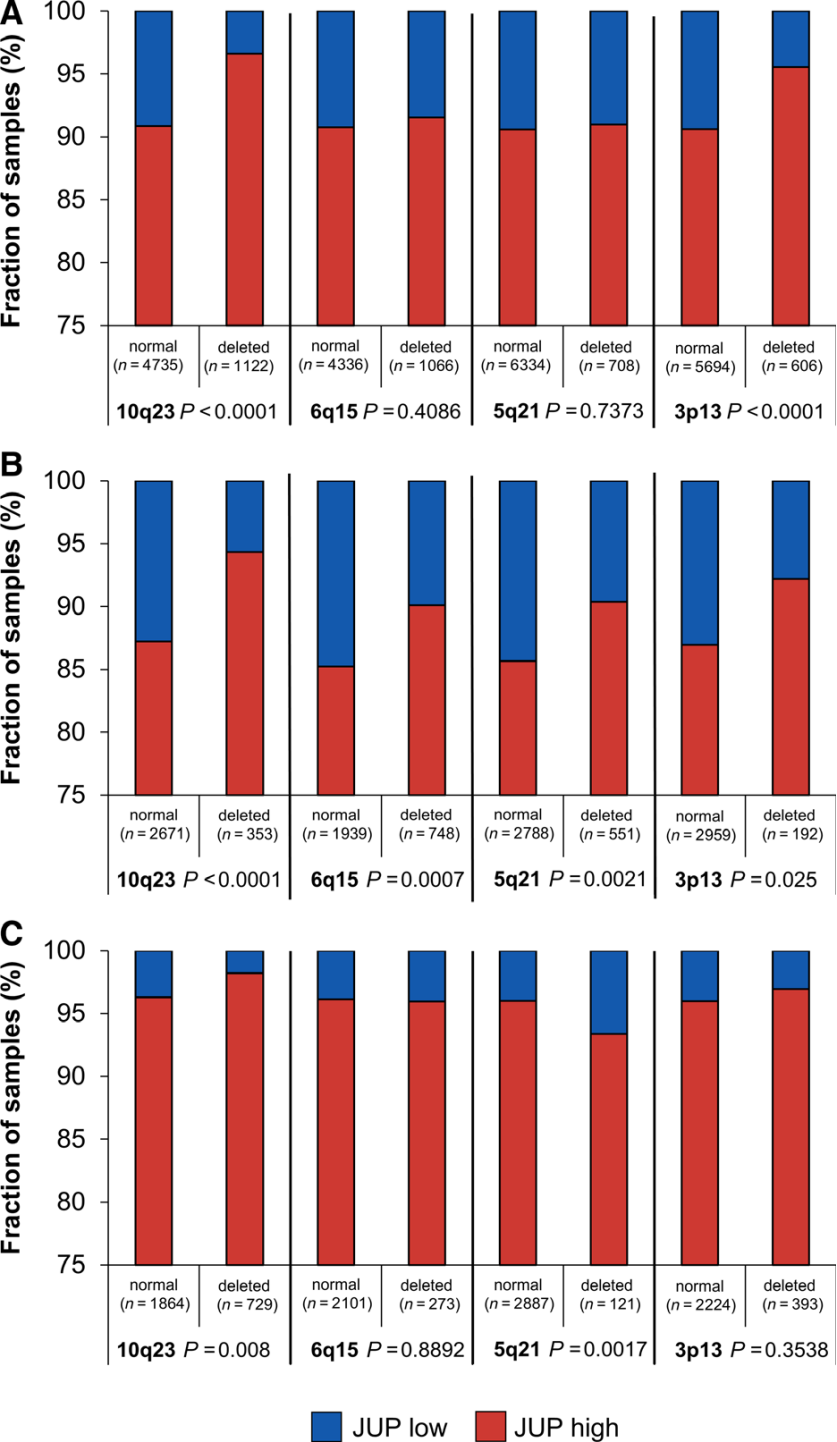
Association between JUP expression and common genomic deletions (10q23, 6q15, 5q21, 3p13) in PCa. (A) All patients, (B) *TMPRSS2:ERG* fusion‐negative patients, (C) *TMPRSS2:ERG* fusion‐positive patients. Association between location of genomic deletion and gene loss in PCa: 10q23—PTEN, 6q15—MAP3K7, 5q21—CHD1, 3p13—FOXP1. For association analysis, contingency tables were used and the chi‐square test was performed to test for statistical significance

Considering all patients, irrespective of the *TMPRSS2:ERG* fusion status, high JUP expression was strongly associated with the 10q23 (*PTEN*) and 3p13 (*FOXP1*) deletion (*P* < 0.0001 each) (Fig. 3A). Interestingly, in the *TMPRSS2:ERG* fusion‐negative subset, high JUP expression was additionally linked to the 6q15 (*MAP3K7*, *P* = 0.0007) and 5q21 (*CHD1*, *P* = 0.0021) deletion while the association with the 3p13 (*FOXP1*) deletion was only weakly detectable (*P* = 0.025; Fig. 3B). In the *TMPRSS2:ERG* fusion‐positive subset, high JUP expression was still associated with the deletion of 10q23 (*PTEN*, *P* = 0.008; Fig. 3C). However, in sharp contrast to the *TMPRSS2:ERG* fusion‐negative subset, in ERG‐positive cancers, there was no association with the *MAP3K7* and *FOXP1* deletion while the *CHD1* deletion was linked to low (and not high) JUP expression (*P* = 0.0017; Fig. 3C).

### 3.5 Association of JUP expression with tumor cell proliferation

High JUP levels were significantly linked to increased cell proliferation, as determined by the Ki67 labeling index (Ki67 Li; Table 4). This association was independent of the Gleason grade as it was significantly detectable in all subgroups of patients with similar Gleason score (≤3 + 3, 3 + 4, 4 + 3, *P* < 0.0001 each; 3 + 4 tertiary 5, *P* = 0.0081; 4 + 3 tertiary 5, *P* = 0.0011; ≥4 + 4 *P* = 0.041; Table 4). The association between high JUP expression and increased cell proliferation was detectable in both *TMPRSS2:ERG* fusion‐positive and *TMPRSS2:ERG* fusion‐negative patients (*P* < 0.0001 each) as well as in the subgroup of patients with normal *CHD1* status (*P* < 0.0001). In the *CHD1*‐deleted subgroup, however, no significant association between JUP and proliferation could be found (*P* = 0.0942).

**Table 4:** Associations between JUP immunostaining results and Ki67 labeling index in all PCa cases, cancers with identical Gleason score, and *ERG* fusion‐negative and *ERG* fusion‐positive cases

Group	JUP	*n*	Ki67 Li (mean)
All cases *P* < 0.0001	low	735	1.42 ± 0.1
	high	5479	2.93 ± 0.04
pGleason ≤ 3+3 *P* < 0.0001	low	219	1.28 ± 0.14
	high	1131	2.36 ± 0.06
pGleason 3 + 4 *P* < 0.0001	low	381	1.39 ± 0.12
	high	3046	2.77 ± 0.04
pGleason 3 + 4 Tert.5 *P* = 0.0081	low	19	1.74 ± 0.56
	high	223	3.3 ± 0.16
pGleason 4 + 3 *P* < 0.0001	low	71	1.55 ± 0.39
	high	534	3.55 ± 0.14
pGleason 4 + 3 Tert.5 *P* = 0.0011	low	23	1.39 ± 0.78
	high	298	4.07 ± 0.22
pGleason ≥ 4+4 *P* = 0.0416	low	22	2.55 ± 0.9
	high	243	4.47 ± 0.27
ERG‐positive *P* < 0.0001	low	136	2.02 ± 0.21
	high	2646	2.91 ± 0.05
ERG‐negative *P* < 0.0001	low	581	1.27 ± 0.11
	high	2731	2.96 ± 0.05
CHD1 deleted *P* = 0.0942	low	44	2.86 ± 0.48
	high	444	3.71 ± 0.15
CHD1 normal *P* < 0.0001	low	371	1.75 ± 0.14
	high	3568	3.03 ± 0.04

### 3.6 Association of JUP expression with AR expression

**Fig. 4:**
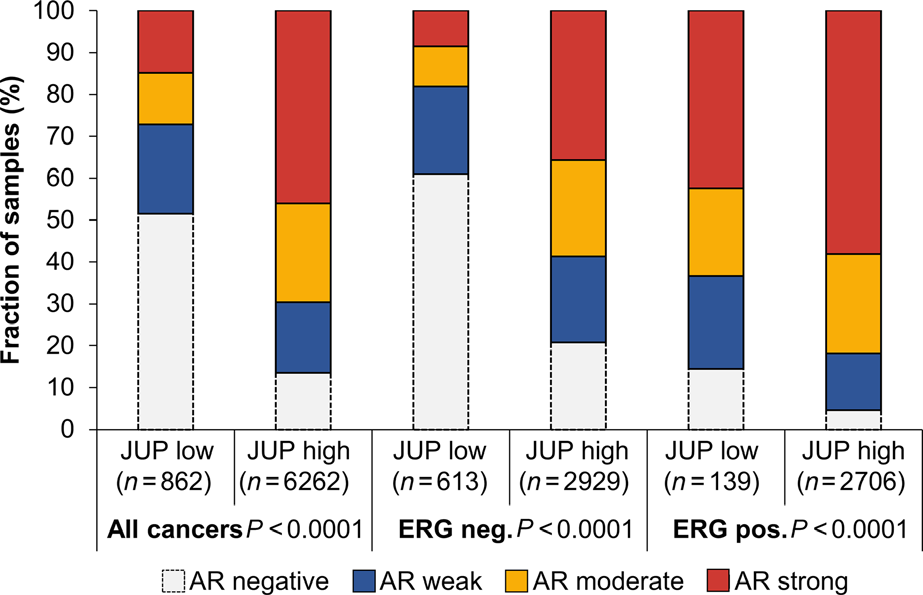
Association between JUP expression and androgen receptor (AR) expression in all PCa cases, *TMPRSS2:ERG* fusion‐negative, and *TMPRSS2:ERG* fusion‐positive subsets. For association analysis, contingency tables were used and the chi‐square test was performed to test for statistical significance

High JUP expression was closely linked to strong AR expression (Fig. 4). Considering all cancers, the fraction of patients with strong AR expression was ~ 15% in the subset of patients with low JUP expression, but ~ 45% in the subset of patients with high JUP expression. Vice versa, about 50% of all patients with low JUP expression showed no AR expression, while only ~ 15% of patients with high JUP expression showed no AR expression. However, the association between high JUP expression and strong AR expression was mainly visible in the subset of ERG‐negative cases. In ERG‐positive cancers, the percentage of patients with strong AR expression was drastically increased despite low JUP levels (Fig. 4).

### 3.7 Association with PSA recurrence (BCR‐free survival)

**Fig. 5:**
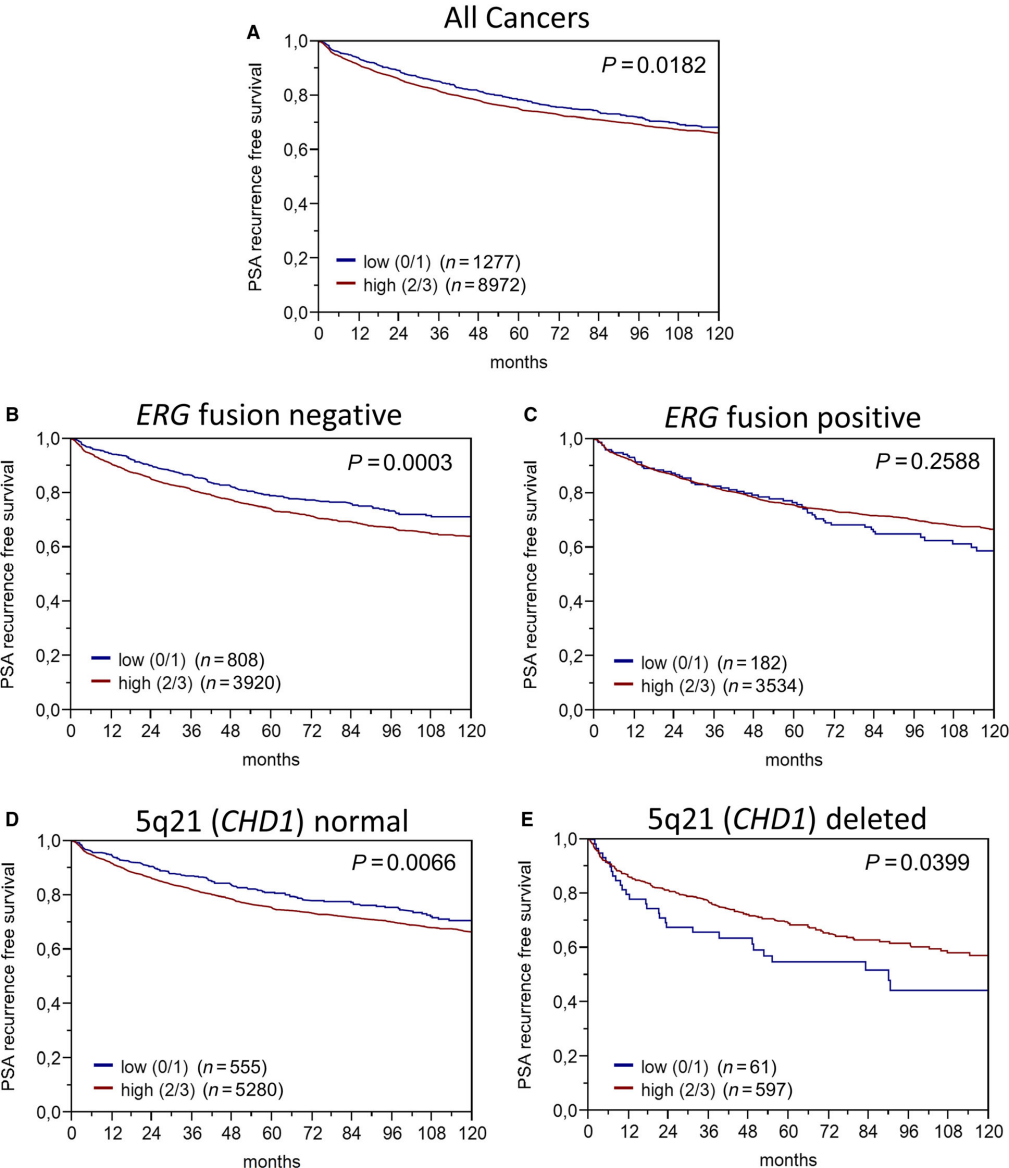
Associations between JUP expression and PSA recurrence. (A) All PCa cases, (B) *TMPRSS2:ERG* fusion‐negative cases, (C) *TMPRSS2:ERG* fusion‐positive cases, (D) 5q21 (*CHD1*) normal cases, and (E) 5q21 (*CHD1*) deleted cases. Time to PSA recurrence (biochemical recurrence (BCR)‐free survival) was defined as the time interval between radical prostatectomy and the first occurrence of postoperative PSA of at least 0.2 ng·ml^−1^ and rising thereafter. Patients without evidence of tumor recurrence were censored at the time of the last follow‐up. BCR‐free survival curves were calculated using the Kaplan–Meier method, and log‐rank test was applied to detect significant differences between groups

Follow‐up data were available from 10 249 patients with interpretable JUP immunostaining on the TMA. Considering all patients, high JUP expression was weakly but significantly linked to early biochemical recurrence following radical prostatectomy (*P* = 0.018; Fig. 5A). Analysis of *JUP* gene expression also found an association with recurrence in the TCGA data set (Fig. S5). We further extended this analysis by studying *JUP* mRNA expression together with adherens junction protein members α‐ or β‐catenin. While, in terms of recurrence, *α‐catenin* and *JUP* expression are positively correlated among each other, *β‐catenin* and *JUP* expression are reciprocally related to each other (Fig. S6).

The association of high JUP protein expression and biochemical recurrence was mainly driven by the subset of *TMPRSS2:ERG* fusion‐negative cancers (*P* = 0.0003; Fig. 5B) and not seen in the *ERG* fusion‐positive subset (*P* = 0.258; Fig. 5C). As the 5q21 (*CHD1*) deletion is mainly occurring in *ERG* fusion‐negative cancers [32], we next analyzed PSA recurrence in the subsets of 5q21‐normal (*n* = 5835) vs. 5q21‐deleted (*n* = 658) patients. Interestingly, while the association between high JUP expression and early PSA recurrence was still detectable in the subset of 5q21‐normal patients (*P* = 0.0066; Fig. 5D), it turned into the opposite in the 5q21‐deleted subset. Here, low JUP expression was linked to early PSA recurrence (*P* = 0.039; Fig. 5E).

### 3.8 Multivariate analyses

Multivariate analyses were performed assessing the clinical relevance of JUP expression in four different scenarios, considering either all patients or patient subsets with the presence or absence of *TMPRSS2:ERG* fusion. Scenario 1 considered all postoperatively available parameters such as preoperative PSA level, pathological tumor stage (pT), pathological Gleason grade based on morphological analysis of the entire prostatectomy specimen, pathological lymph node status (pN), surgical margin status (R), and JUP expression (overall 6521 samples analyzable with 3033 of them with known ERG‐negative and 2363 with known ERG‐positive status; Table 5). In scenario 2, JUP was tested against the same parameters as in scenario 1 except nodal status. The reason for excluding nodal status was that both indication and degree of lymph node dissection are not standardized in the surgical therapy of PCa and that excluding pN in multivariate analyses can notably increase the number of analyzable cases (resulting in overall 10 211 analyzable cases in scenario 2). Two additional scenarios aimed at modeling the preoperative situation as closely as possible. Scenario 3 included JUP expression, preoperative PSA, clinical tumor stage (cT), and Gleason grade obtained on the prostatectomy specimens. Since postoperative assessment of a tumor’s Gleason grade is superior to the preoperatively determined Gleason grade [41], we added scenario 4, in which the preoperative Gleason grade obtained from biopsy specimens was combined with preoperative PSA, cT stage, and JUP expression. These analyses revealed that JUP expression is an independent prognostic factor in all pre‐ and postsurgical scenarios, when considering the subset of *TMPRSS2:ERG* fusion‐negative cases (*P* < 0.05 each). When considering all patients, JUP was only independently predictive of early PSA recurrence in scenario 4 (*P* = 0.009). When considering the ERG‐positive patient subset, JUP was not independent of established prognostic markers irrespective of the scenario. All scenarios are summarized in Table 5 (for further details on *P*‐values and hazard ratios for all comparisons, see Table S5).

**Table 5:** Multivariate Cox regression analysis, including established prognostic parameters and JUP expression, in all PCa cases and the *TMPRSS2: ERG* fusion‐negative and *TMPRSS2: ERG* fusion‐positive subset

	Scenario	*n* analyzable	*P*‐value							
			Preoperative PSA level	pT stage	cT stage	Gleason grade prostatectomy	Gleason grade biopsy	pN stage	R stage	JUP
All cancers	1	6521	<0.0001	<0.0001	—	<0.0001	—	<0.0001	<0.0001	0.1681
	2	10 211	<0.0001	<0.0001	—	<0.0001	—	—	<0.0001	0.4412
	3	10 073	<0.0001	—	<0.0001	<0.0001	—	—	—	0.2635
	4	9382	<0.0001	‐	<0.0001	‐	<0.0001	—	—	0.0097
ERG‐negative	1	3033	0.0003	<0.0001	—	<0.0001	—	<0.0001	0.2792	0.0054
	2	4713	<0.0001	<0.0001	—	<0.0001	—	—	0.0067	0.0462
	3	4666	<0.0001	—	<0.0001	<0.0001	—	—	—	0.0483
	4	4590	<0.0001	—	<0.0001	‐	<0.0001	—	—	0.0097
ERG‐positive	1	2363	0.0135	<0.0001	—	<0.0001	—	0.0221	0.0001	0.1196
	2	3702	<0.0001	<0.0001	—	<0.0001	—	—	<0.0001	0.0954
	3	3639	<0.0001	—	<0.0001	<0.0001	—	—	—	0.1385
	4	3583	<0.0001	—	<0.0001	—	<0.0001	—	—	0.6876

### 3.9 Associations of JUP and ERG with WNT target gene expression

As the overall adverse prognostic effect of high JUP expression was absent in *TMPRSS2:ERG* fusion‐positive patients (see 3.8) and as both ERG and JUP are involved in the regulation of WNT signaling, we further checked the influence of the ERG and JUP status on WNT target gene expression by means of *AXIN2*, *NKD1*, *LEF1,* and *MYC* (Fig. S4). Among these, a positive correlation of *ERG* expression only with *AXIN2* and *LEF1* expression was found while JUP *per se* was negatively correlated with expression of *AXIN2*, *NKD1*, and *LEF1*. Interestingly, this negative correlation got lost in ERG‐positive patients in case of *AXIN2*. In addition, *JUP* was significantly inversely correlated with *MYC* expression specifically in the ERG‐negative subset.

## 4 Discussion

Studies exploring the role of JUP in PCa are rare and were mainly focused on the differential expression of JUP in normal prostate versus prostate cancer tissue [24, 25, 26]. These data suggest that JUP expression is reduced in PCa as compared to normal tissue samples; the expression of JUP in different molecular subsets, however, has not been assessed so far and might be diverse.

Among known molecular markers, which in part harbor their own prognostic relevance or significantly contribute to the pathophysiology of PCa, the *TMPRSS2:ERG* gene fusion is the most frequent. Importantly, ERG was shown to be an inducer of β‐catenin‐dependent WNT/LEF1 signaling and target gene expression in PCa [42], which in turn may be regulated by JUP. Therefore, it was of great interest to analyze the clinical impact of JUP in the *ERG* fusion‐negative vs. *ERG* fusion‐positive subsets. Interestingly, the association of high JUP expression with adverse tumor features was mainly observed in *ERG* fusion‐negative patients. Accordingly, the outcome of ERG‐negative patients with high JUP expression was significantly worse compared with those with low JUP expression. The prognostic impact of this association was statistically independent of established prognostic parameters as confirmed by multivariate analyses.

In contrast, JUP expression had no significant prognostic effect in the ERG‐positive subset in uni‐ or multivariate analyses. This observation was most likely due to the notable increase in JUP expression in the ERG‐positive fraction, suggesting JUP as a putative ERG target gene. From a statistical point of view, the imbalanced distribution of JUP low *vs*. high patients in the ERG‐positive subset (4.7 *vs*. 95.3%) might have precluded an outcome difference. Moreover, high JUP levels were linked to all tested common deletions (*PTEN, CHD1, MAP3K7* *, FOXP1*) in the ERG‐negative, but not in the ERG‐positive subset (Fig. S7) and all these deletions are known to indicate reduced BCR‐free survival in PCa [32, 33, 34, 35]. In addition, the difference in the Ki67 proliferation index between JUP low vs. high patients was relatively small (albeit significant) in the ERG‐positive (2.02 vs. 2.91) as compared to the ERG‐negative subset (1.27 vs. 2.96). High Ki67 proliferation index is an independent predictor of tumor progression in PCa [43]. Other reasons might be competing effects of JUP and ERG on WNT signaling. Based on our TCGA analysis, *JUP* appears to reduce WNT target gene expression either in the overall PCa cohort (*AXIN2*, *LEF1*, *NKD1*) or specifically in ERG‐negative patients (*MYC*) while *ERG* fusion *per se* apparently induces WNT signaling to some extent (*AXIN2*, *LEF1*, but not *NKD1* or *MYC*). However, the inverse correlation of *JUP* and WNT target genes gets only partially lost in the ERG fusion‐positive subset (*AXIN2*, *MYC*). Therefore, it would be highly speculative to explain the lack of prognostic relevance of JUP in the ERG‐positive subset by an overriding effect of ERG on WNT signaling. Instead, it can also be hypothesized that JUP positively influenced AR expression specifically in the ERG‐negative subset (encouraging further research on a putative functional interaction of JUP with AR in the absence of *ERG*) and high AR levels are known to be unfavorable [44]. Nevertheless, taking our *in silico* (TCGA) and TMA findings together, our study suggests that the unfavorable effect of high JUP levels in ERG‐negative patients is accompanied by decreased WNT signaling. Hence, the effects of WNT signaling on PCa progression might be diverse depending on the exact context [42, 45, 46].

Earlier studies identified chromosomal deletions that were predominantly found either in *ERG* fusion‐positive (*FOXP1, PTEN*) or *ERG* fusion‐negative PCa (*CHD1, MAP3K7*) [32, 33, 34, 35]. These associations were also found in the expanded cohorts of the present study. Surprisingly, while high JUP expression is unfavorable in the ERG‐negative subset, low JUP expression is unfavorable in the *CHD1*‐deleted subset although the majority of *CHD1*‐deleted patients are ERG‐negative. Thus, inversely to the ERG‐negative cohort itself, low JUP expression was associated with early PSA recurrence after RP in the *CHD1*‐deleted subset. *CHD1* deletion is strongly linked to poor patient outcomes [32] and predicts shortened metastasis‐free survival after RP in R0 patients [47]. Also, CHD1 protein loss in conjunction with MAP3K7 loss correlates with decreased E‐cadherin expression in clinical samples [48].

Here, loss of JUP, presumably contributing to reduced tumor cell cohesion by disturbing desmosome and adherens junction assembly, might additionally promote the more metastasis‐prone phenotype of *CHD1*‐deleted cancers within the ERG‐negative subset. This effect might override the general proproliferative effect of high JUP expression. Intriguingly, the association between high JUP and high Ki67 was specifically absent in the *CHD1*‐deleted subset of patients compared with all other subsets. This hypothesis is also supported by a study from Franzen *et al*., demonstrating that experimental downregulation of JUP expression in PCa cell lines leads to a substantial weakening of cell–cell adhesion and an EMT‐like phenotype [49]. In addition, we recently published that the gene and protein expression levels of desmosomal and adherens junction proteins such as JUP, DSP, DSG2, CDH1, and CTNNA1 are decreased with rising metastatic potential in spontaneous metastasis xenograft models of human PCa [50]. Interestingly, our analysis of *JUP* gene expression in published GEO data sets demonstrated that, on the one hand, *JUP* expression is increased in localized prostate tumors compared with normal tissue, but, on the other hand, is decreased in PCa metastasis compared with localized tumors. Finally, the notion, that loss of JUP expression favors metastasis, is also supported by *in vitro* studies in other entities [11, 51, 52, 53].

This study has potential limitations. It remains unclear why the prognostic role of JUP turns into the opposite in the *CHD1*‐deleted subset. We can only speculate that this might be related to a different functional role of JUP in the more metastasis‐prone phenotype of *CHD1*‐deleted PCa cells. Further experiments should be conducted to explore the functional consequences of JUP depletion in *CHD1*‐normal vs. *CHD1*‐depleted PCa cells or xenograft models.

## 5 Conclusions

In conclusion, this study extends previous insights into the role of JUP in PCa on the clinical level in a large‐enough patient cohort to reflect the molecular heterogeneity of the disease. The opposing biological roles of JUP are reflected by antagonistic prognostic effects in different molecular subtypes. Specifically, high JUP expression was associated with more proliferation and an unfavorable outcome in the *CHD1*‐normal and overall cohort; in contrast, JUP was not linked to proliferation in *CHD1*‐deleted patients, where low JUP expression was unfavorable. Further studies on the divergent role of JUP in PCa are encouraged by these findings.

## Conflict of interest

The authors declare no conflict of interest.

## Author contributions

TS performed experiments and wrote the manuscript; LCB analyzed data and aided in writing the manuscript; VL analyzed data and reviewed the manuscript; AKA analyzed data and reviewed the manuscript; JSS performed experiments; SB performed experiments; RS analyzed data; GS provided resources; HH provided resources; RK analyzed data and reviewed the manuscript; US provided resources and reviewed the MS; TL analyzed data, supervised the study, and reviewed the MS.

## Supporting information


**Table S1.** Association between ERG immunostaining results and PCa phenotype in all cancers.
**Table S2.** Associations between trichotomized JUP immunostaining results and PCa phenotype in all cases analyzed.
**Table S3.** Associations between trichotomized JUP immunostaining results and PCa phenotype in the TMPRSS2:ERG fusion‐negative subset.
**Table S4.** Associations between trichotomized JUP immunostaining results and PCa phenotype in the TMPRSS2:ERG fusion‐positive subset.
**Table S5.** Multivariate Cox regression analysis, including p‐values and hazard ratios for all comparisons.
**Fig. S1.** Representative images of JUP immunostaining in cancerous prostate tissue.
**Fig. S2.** Associations between JUP expression (trichotomized IHC staining tissue.
**Fig. S3.** Correlation of JUP gene expression with ERG in PCa (TCGA data set), but not in benign/normal prostate gland (TCGA/GTEx combined data).
**Fig. S4.** Influence of JUP on Wnt target gene expression in the TCGA prostate cancer data set analyzed using cBioPortal.
**Fig. S5.** JUP gene expression and disease‐free survival in the TCGA prostate cancer data set analyzed using cBioPortal.
**Fig. S6.** Combined analysis of JUP gene expression along with α‐catenin or β‐catenin gene expression for disease‐free survival in the TCGA prostate cancer data set using cBioPortal.
**Fig. S7.** Genomic aberrations and JUP status (graphical abstract).
