# Supplementary material for: Opposing prognostic relevance of junction plakoglobin in distinct prostate cancer patient subsets
Source: Mol Oncol. 2021 Feb 17;15(7):1956–69. doi: 10.1002/1878-0261.12922 (PMC8253102; doi:10.1002/1878-0261.12922)

Supplementary Data

Table S1: Association between ERG immunostaining results and prostate cancer phenotype in all cancers.

Parameter	n evaluable	low (0/1) %	high (2/3) %	*p*-value
**All Cancers**	10,880	56.3	43.7	
**Tumor Stage**				
pT2	7,034	58.9	41.1	
pT3a	2,432	49.8	50.2	<0.0001
pT3b-pT4	1,378	54.6	45.4	
**Gleason Grade**				
≤3+3	2,232	56.5	43.5	
3+4	5,979	54.0	46.0	
3+4 Tert.5	400	65.3	34.8	<0.0001
4+3	1,101	58.6	41.4	
4+3 Tert.5	626	59.3	40.7	
≥4+4	534	65.5	34.5	
**Quantitative Gleason Grade**				
≤3+3	2,232	56.5	43.5	
3+4 ≤5%	1,584	55.6	44.4	
3+4 6-10%	1,579	53.5	46.5	
3+4 11-20%	1,304	55.3	44.7	
3+4 21-30%	696	51.0	49.0	
3+4 31-49%	560	56.4	43.6	<0.0001
3+4 Tert.5	400	65.3	34.8	
4+3 50-60%	473	57.5	42.5	
4+3 Tert.5	626	59.3	40.7	
4+3 61-100%	466	67.2	32.8	
≥4+4	515	60.2	39.8	
**Lymph Node Metastasis**				
N0	6,272	56.6	43.4	0.109
N+	636	53.3	46.7	
**Preoperative PSA Level (ng/ml)**				
<4	1,340	49.7	50.3	
4-10	6,555	55.3	44.7	<0.0001
10-20	2,170	61.3	38.7	
>20	750	63.2	36.8	
**Surgical Margin**				
negative	8,685	56.6	43.4	0.2344
positive	2,160	55.2	44.8	
**Age at time of surgery**				
<50	308	41.9	58.1	
50-60	2,691	50.8	49.2	<0.0001
60-70	6,279	57.9	42.1	
>70	1,563	62.4	37.6	

Table S2: Associations between trichotomized JUP immunostaining results and PCa phenotype in all cases analyzed.

Parameter	n evaluable	low (0/1) %	medium (2) %	high (3) %	*p*-value
**All Cancers**	11,267	12.4	50.6	37.0	
**Tumor Stage**					
pT2	7,285	13.1	50.2	36.7	0.0014
pT3a	2,486	12.1	50.2	37.7	
pT3b-pT4	1,455	9.3	53.1	37.5	
**Gleason Grade**					
≤3+3	2,388	15.8	52.5	31.7	<0.0001
3+4	6,066	12.5	48.8	38.8	
3+4 Tert.5	434	8.5	52.8	38.7	
4+3	1,114	11.0	52.2	36.8	
4+3 Tert.5	690	8.4	52.0	39.6	
≥4+4	566	8.1	55.8	36.0	
**Quantitative Gleason Grade**					
≤3+3	2,388	15.8	52.5	31.7	<0.0001
3+4 ≤5%	1,627	13.3	49.4	37.3	
3+4 6-10%	1,583	12.4	45.8	41.8	
3+4 11-20%	1,305	12.0	46.7	41.4	
3+4 21-30%	691	10.6	50.9	38.5	
3+4 31-49%	575	11.5	50.6	37.9	
3+4 Tert.5	434	8.5	52.8	38.7	
4+3 50-60%	489	12.1	48.9	39.1	
4+3 Tert.5	690	8.4	52.0	39.6	
4+3 61-100%	505	7.7	55.2	37.0	
≥4+4	508	8.5	53.9	37.6	
**Lymph Node Metastasis**					
N0	6,500	12.9	50.3	36.7	0.0403
N+	692	9.7	52.3	38.0	
**Preoperative PSA Level (ng/ml)**					
<4	1,410	8.9	48.9	42.1	<0.0001
4-10	6,754	12.2	50.2	37.6	
10-20	2,245	14.7	51.4	34.0	
>20	783	13.2	55.2	31.7	
**Surgical Margin**					
negative	8,989	12.5	50.5	36.9	0.5571
positive	2,237	11.7	51.0	37.3	
**Age at time of surgery**					
<50	293	9.6	50.5	39.9	0.0805
50-60	2,815	11.6	50.0	38.4	
60-70	6,488	12.5	50.6	36.9	
>70	1,627	13.7	51.7	34.6	

Table S3: Associations between trichotomized JUP immunostaining results and PCa phenotype in the *TMPRSS2:ERG* fusion-negative subset.

Parameter	n evaluable	low (0/1) %	medium (2) %	high (3) %	*p*-value
**All Cancers**	5,124	16.9	53.9	29.1	
**Tumor Stage**					
pT2	3,431	17.7	54.3	28.0	
pT3a	1,024	17.2	52.8	30.0	0.0012
pT3b-pT4	655	12.2	53.7	34.0	
**Gleason Grade**					
≤3+3	1,021	23.6	56.1	20.3	
3+4	2,692	17.1	52.2	30.8	
3+4 Tert.5	227	8.8	61.2	30.0	<0.0001
4+3	550	15.5	54.9	29.6	
4+3 Tert.5	321	9.7	53.3	37.1	
≥4+4	309	9.7	56.0	34.3	
**Quantitative Gleason Grade**					
≤3+3	1,021	23.6	56.1	20.3	
3+4 ≤5%	712	18.3	53.9	27.8	
3+4 6-10%	707	17.7	52.1	30.3	
3+4 11-20%	601	15.1	49.8	35.1	
3+4 21-30%	304	16.8	50.7	32.6	
3+4 31-49%	267	15.4	52.4	32.2	<0.0001
3+4 Tert.5	227	8.8	61.2	30.0	
4+3 50-60%	234	18.4	50.9	30.8	
4+3 Tert.5	321	9.7	53.3	37.1	
4+3 61-100%	280	8.9	55.4	35.7	
≥4+4	262	11.1	58.8	30.2	
**Lymph Node Metastasis**					
N0	2,992	16.7	52.5	30.7	0.0053
N+	298	11.7	49.3	38.9	
**Preoperative PSA Level (ng/ml)**					
<4	547	13.5	51.2	35.3	
4-10	3,018	17.0	54.0	29.0	0.0186
10-20	1,125	18.2	55.0	26.8	
>20	407	16.5	55.0	28.5	
**Surgical Margin**					
negative	4,093	17.1	54.3	28.6	0.2255
positive	1,018	16.1	52.6	31.3	
**Age at time of surgery**					
<50	104	11.5	61.5	26.9	
50-60	1,152	17.2	52.0	30.8	0.4683
60-70	3,059	16.9	54.3	28.8	
>70	794	17.3	54.3	28.5	

Table S4: Associations between trichotomized JUP immunostaining results and PCa phenotype in the *TMPRSS2:ERG* fusion-positive subset.

Parameter	n evaluable	low (0/1) %	medium (2) %	high (3) %	*p*-value
**All Cancers**	4,052	4.7	45.1	50.1	
**Tumor Stage**					
pT2	2,419	4.4	44.1	51.5	
pT3a	1,072	5.3	45.8	48.9	0.2227
pT3b-pT4	544	4.2	48.5	47.2	
**Gleason Grade**					
≤3+3	843	6.6	49.8	43.5	
3+4	2,321	4.4	43.5	52.0	
3+4 Tert.5	123	4.1	36.6	59.3	
4+3	394	4.1	46.4	49.5	0.0006
4+3 Tert.5	220	1.8	46.8	51.4	
≥4+4	148	4.7	45.9	49.3	
**Quantitative Gleason Grade**					
≤3+3	843	6.6	49.8	43.5	
3+4 ≤5%	585	3.8	43.1	53.2	
3+4 6-10%	612	3.9	37.7	58.3	
3+4 11-20%	501	4.2	42.1	53.7	
3+4 21-30%	293	3.4	46.8	49.8	
3+4 31-49%	208	6.7	45.2	48.1	<0.0001
3+4 Tert.5	123	4.1	36.6	59.3	
4+3 50-60%	177	4.0	41.2	54.8	
4+3 Tert.5	220	1.8	46.8	51.4	
4+3 61-100%	123	4.1	43.1	52.8	
≥4+4	174	2.3	47.7	50.0	
**Lymph Node Metastasis**					
N0	2,316	5.0	45.8	49.2	0.1521
N+	251	6.4	50.6	43.0	
**Preoperative PSA Level (ng/ml)**					
<4	553	3.1	45.8	51.2	
4-10	2,494	4.4	44.9	50.7	0.0317
10-20	722	5.7	43.5	50.8	
>20	253	6.7	51.4	41.9	
**Surgical Margin**					
negative	3,180	4.8	44.7	50.5	0.4990
positive	854	4.1	46.6	49.3	
**Age at time of surgery**					
<50	149	7.4	42.3	50.3	
50-60	1,149	4.9	47.0	48.1	0.4120
60-70	2,264	4.4	44.8	50.8	
>70	471	4.2	42.9	52.9	

Table S5: Multivariate Cox regression analysis, including *p*-values and hazard ratios for all comparisons.

	Scenario 1			Scenario 2			Scenario 3			Scenario 4		
	**All cancers**	**ERG neg.**	**ERG pos.**	**All cancers**	**ERG neg.**	**ERG pos.**	**All cancers**	**ERG neg.**	**ERG pos.**	**All cancers**	**ERG neg.**	**ERG pos.**
**n analyzable**	6,521	3,033	2,363	10,211	4,713	3,702	10,073	4,666	3,639	9,382	4,59	3,583
**Gleason grade biopsy**										[***]	[***]	[***]
3+4 vs. ≤3+3										1.84***	1.6***	1.98***
4+3 vs. 3+4										1.78***	1.76***	1.77***
≥4+4 vs. 4+3										1.27**	2.19***	1.41**
**cT Stage**							[***]	[***]	[***]	[***]	[***]	[***]
T2a vs. T1c							1.25***	1.28*	1.07*	1.26***	1.27*	1.32**
T2b vs. T2a							1.38***	1.6***	1.18	1.53***	1.65***	1.37*
T2c vs. T2b							1.14	0.88	1.26	1.08	0.95	1.13
T3a vs. T2c							0.87	0.88	1.05	0.74	0.66	0.88
**preoperative PSA-Level**	[***]	[**]	[*]	[***]	[***]	[***]	[***]	[***]	[***]	[***]	[***]	[***]
4-10 vs. <4	1.16	1.01	1.34*	1.21*	0.97	1.43*	1.33**	1.06	1.68***	1.43***	1.09	1.82***
10-20 vs. 4-10	1.19*	1.23*	1	1.29***	1.34***	1.1	1.43***	1.47***	1.22*	1.53***	1.6***	1.36**
>20 vs. 10-20	1.2*	1.2	1.26*	1.19*	1.16	1.25*	1.37***	1.28*	1.54**	1.64***	1.59***	1.63***
**JUP expression**	[n.s.]	[*]	[n.s]	[n.s.]	[*]	[n.s.]	[n.s.]	[*]	[n.s.]	[*]	[**]	[n.s.]
low vs. high	0.91	**0.77***	1.27	0.95	**0.85***	1.26	0.93	**0.85***	1.23	**0.85***	**0.76****	1.06
**Gleason grade prostatectomy**	[***]	[***]	[***]	[***]	[***]	[n.s.]	[***]	[***]	[***]			
3+4 vs. ≤3+3	2.14***	1.81***	2.37***	2.31***	2.43***	2.37***	2.84***	2.38***	2.93***			
4+3 vs. 3+4	1.96***	1.95***	1.87***	2.12***	1.96***	2.15***	2.56***	2.17***	2.86***			
≥4+4 vs. 4+3	1.31**	1.27*	1.67**	1.38***	1.31*	1.85***	1.92***	1.85***	2.32***			
**pT-Stage**	[***]	[***]	[***]	[***]	[***]	[***]						
T3a vs. T2	1.97***	1.86***	2.31***	1.94***	1.84***	2.11***						
T3b vs. T3a	1.48***	1.46***	1.47***	1.67***	1.67***	1.6***						
T4 vs. T3b	1.26	1.07	1.46	1.21	1.08	1.38						
**R-Stage**	[***]	[n.s.]	[***]	[***]	[*]	[***]						
R1 vs. R0	1.24***	1.09	1.37***	1.37***	1.21*	1.47***						
**N-Stage**	[***]	[***]	[*]									
N+ vs. N0	1.5***	1.52***	1.28*									

*p≤0.05, **p≤0.001, ***p≤0.0001, [ ]=over all *p*-value

Figure S1: Representative images of JUP immunostaining in cancerous prostate tissue. Panel (A): Negative JUP expression. Panel (B): low JUP expression. Panel (C): high JUP expression. Scale bar = 100 μm.

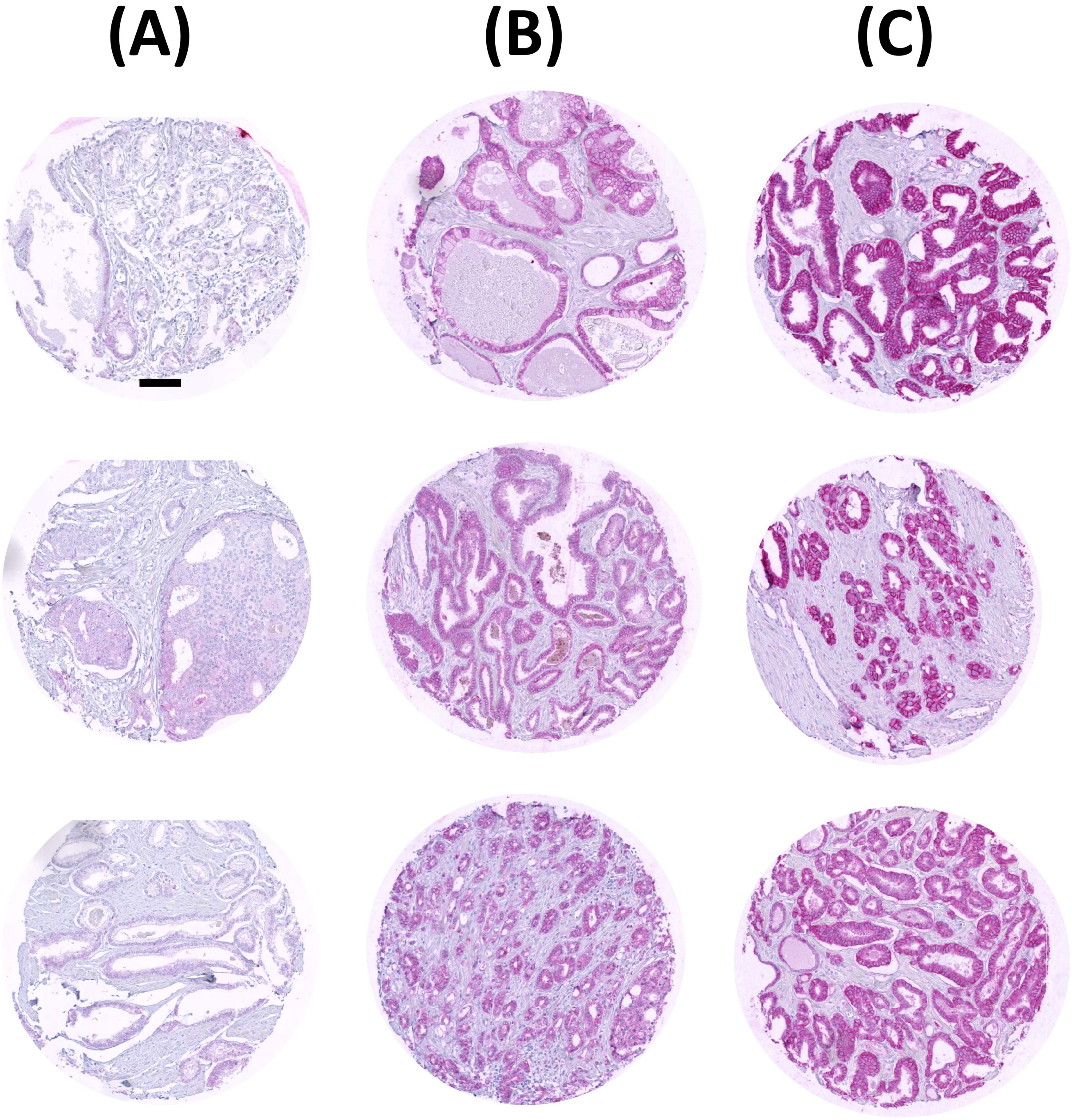

Figure S2: Associations between JUP expression (trichotomized IHC staining intensity) and PSA recurrence. (A) All PCa cases, (B) *TMPRSS2:ERG* fusion-negative cases, (C) *TMPRSS2:ERG* fusion-positive cases, (D) 5q21 (*CHD1*) normal cases, (E) 5q21 (*CHD1*) deleted cases. Time to PSA recurrence (biochemical recurrence (BCR)-free survival) was defined as the time interval between radical prostatectomy and the first occurrence of post-operative PSA of at least 0.2 ng/ml and rising thereafter. Patients without evidence of tumor recurrence where censored at the time of the last follow-up. BCR-free survival curves were calculated using the Kaplan-Meier method and Log-Rank test was applied to detect significant differences between groups, as indicated in the graph.

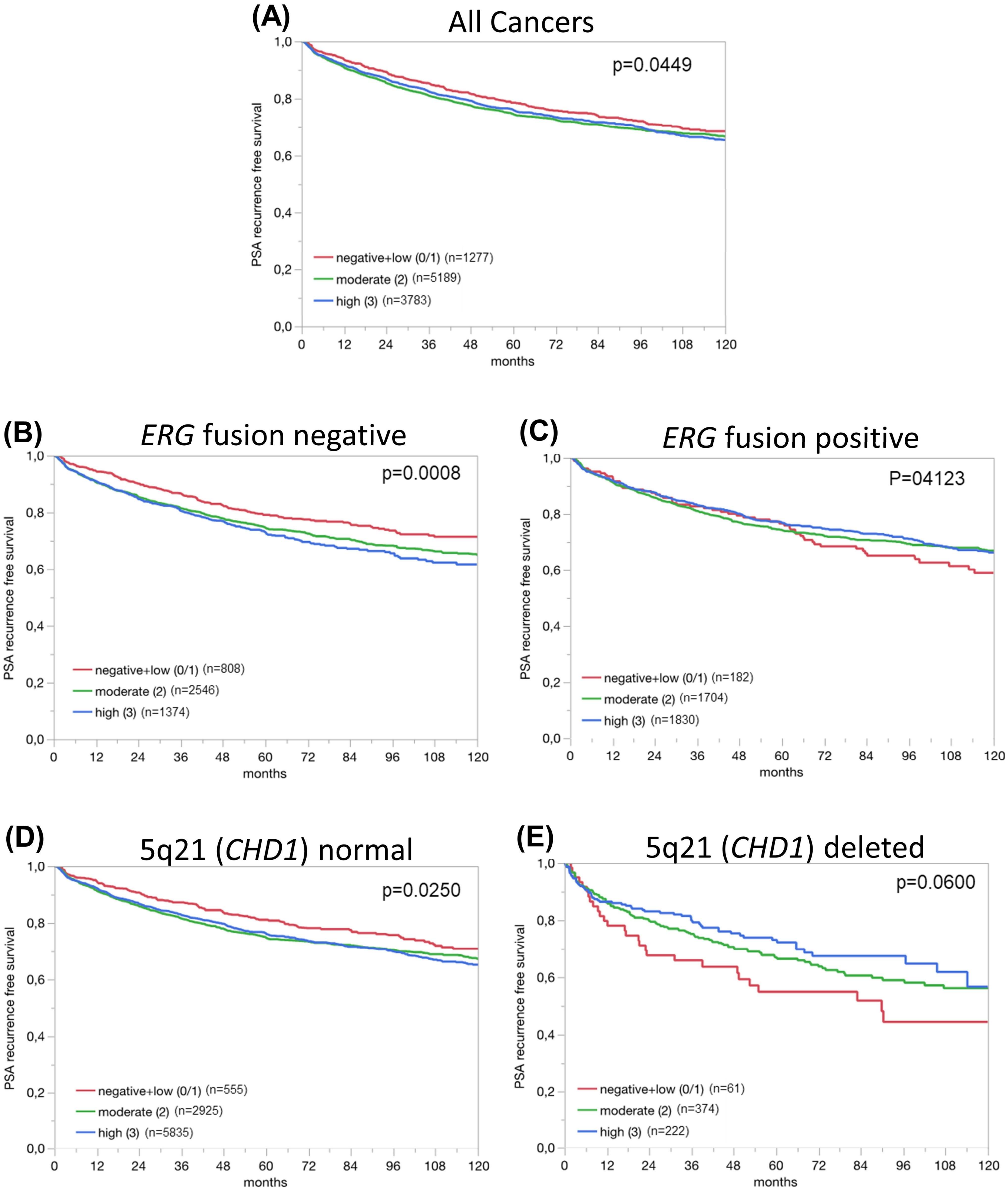
(A)
All Cancers
p=0.0449
PSA recurrence free survival
negative+low (0/1) (n=1277)
moderate (2) (n=5189)
high (3) (n=3783)
months
(B)
ERG fusion negative
p=0.0008
PSA recurrence free survival
negative+low (0/1) (n=808)
moderate (2) (n=2546)
high (3) (n=1374)
months
(C)
ERG fusion positive
P=04123
PSA recurrence free survival
negative+low (0/1) (n=182)
moderate (2) (n=1704)
high (3) (n=1830)
months
(D)
5q21 (CHD1) normal
p=0.0250
PSA recurrence free survival
negative+low (0/1) (n=555)
moderate (2) (n=2925)
high (3) (n=5835)
months
(E)
5q21 (CHD1) deleted
p=0.0600
PSA recurrence free survival
negative+low (0/1) (n=61)
moderate (2) (n=374)
high (3) (n=222)
months

Figure S3: Correlation of JUP gene expression with ERG in PCa (TCGA data set), but not in benign/normal prostate gland (TCGA/GTEx combined data). Pearson correlation analysis was used to determine statistical significance, as indicated in the graph. n(PCa)=492, n(Normal)=152.

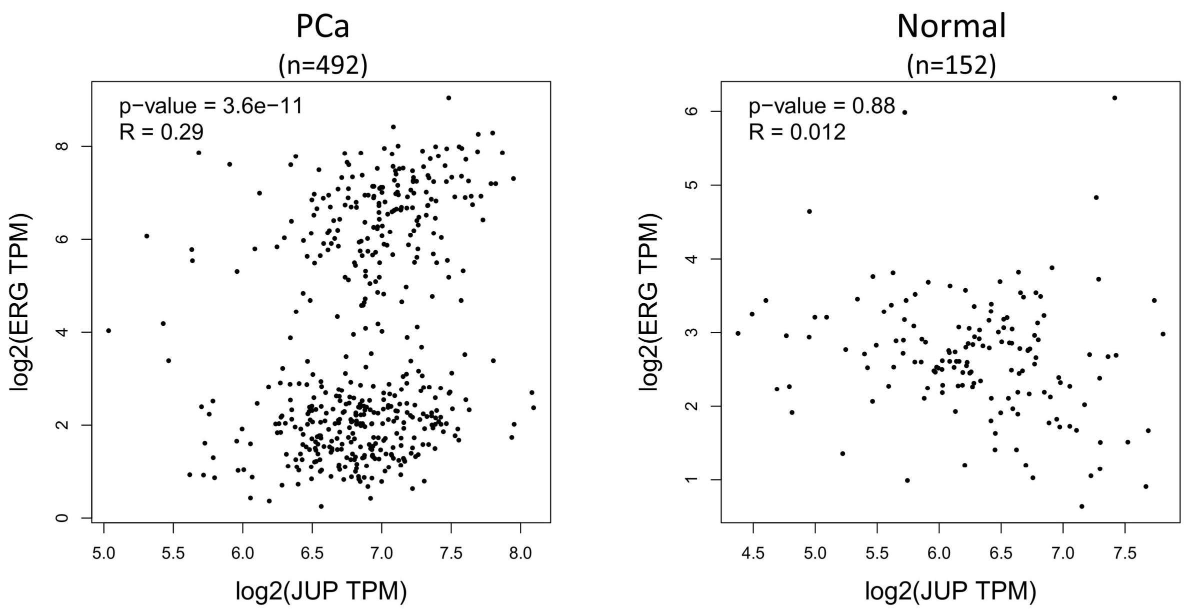

Figure S4: Influence of JUP on Wnt target gene expression. The TCGA data set was analyzed for mRNA expression of Wnt target genes *AXIN2, NKD1, LEF1, and MYC* using cBioPortal, stratifying patients for JUP mRNA expression and ERG fusion status. q values were calculated on cBioPortal using Student's t-test of 2-sample with equal or unequal variance and Benjamini-Hochberg correction. *q<0.05.

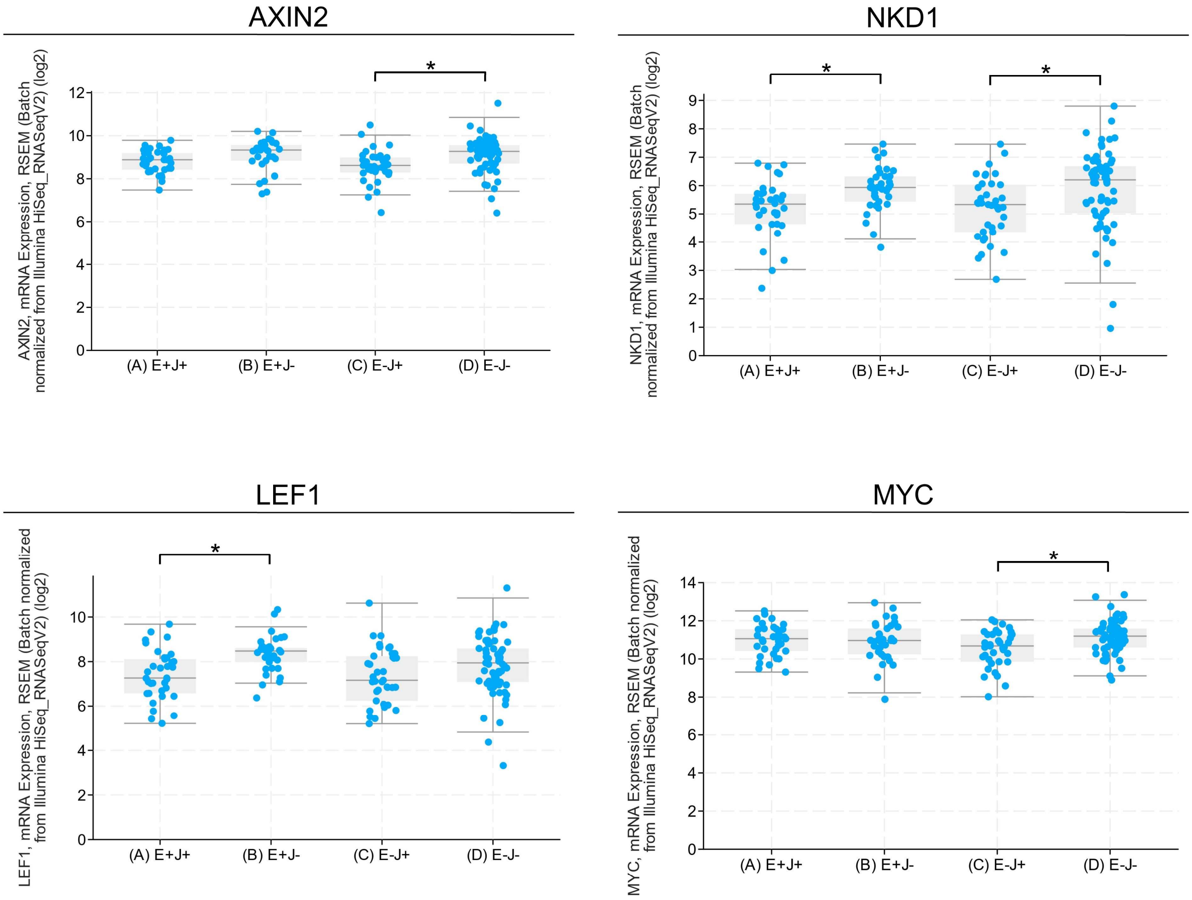

	JUP- (n= 98)	JUP+ (n= 72)	q-value	ERG- (n=291)	ERG+ (n=203)	q-value
**JUP**	12.26	13.3	**8.08e-87**	12.74	12.82	**0.0153**
**ERG**	9.63	10.01	0.442	7.71	12.34	**3.74e-173**
**AXIN2**	9.11	8.7	**0.001**	8.94	9.12	**0.0132**
**NKD1**	5.84	5.14	**0.0009**	5.63	5.62	0.942
**LEF1**	8.01	7.38	**0.0025**	7.53	7.95	**0.00021**
**MYC**	11.05	10.76	0.0779	10.99	11.13	0.15

	E+/J+ (n=35)	E+/J- (n=35)	E-/J+ (n=37)	E-/J- (n=63)	q-value (E+/J- vs. E+/J+)	q-value (E/J- vs. E-/J+)
**JUP**	13.34	12.26	13.26	12.26	**3.56e-35**	**1.45e-49**
**ERG**	12.71	12.49	7.45	8.04	0.418	**0.0049**
**AXIN2**	8.81	9.12	8.6	9.11	0.094	**0.0131**
**NKD1**	5.14	5.88	5.15	5.82	**0.008041**	**0.0491**
**LEF1**	7.4	8.35	7.36	7.82	**0.001493**	0.173
**MYC**	10.98	10.92	10.55	11.12	0.853	**0.0209**

Figure S5: *JUP* gene expression and disease-free survival in the TCGA prostate cancer data set analyzed using cBioPortal. JUP mRNA expression z-scores were compared using a threshold ± 1 to define high and low expression, compared to expression in all the tumors diploid for JUP. Using this threshold, 35% of patient tumors were classified as high or low JUP. Recurrence was observed in 12/52 patients with high JUP and in 4/67 patients with low JUP expression. Disease-free survival curves were calculated using the Kaplan-Meier method and Log-Rank test was applied to detect significant differences between groups (*p*=0.009496).

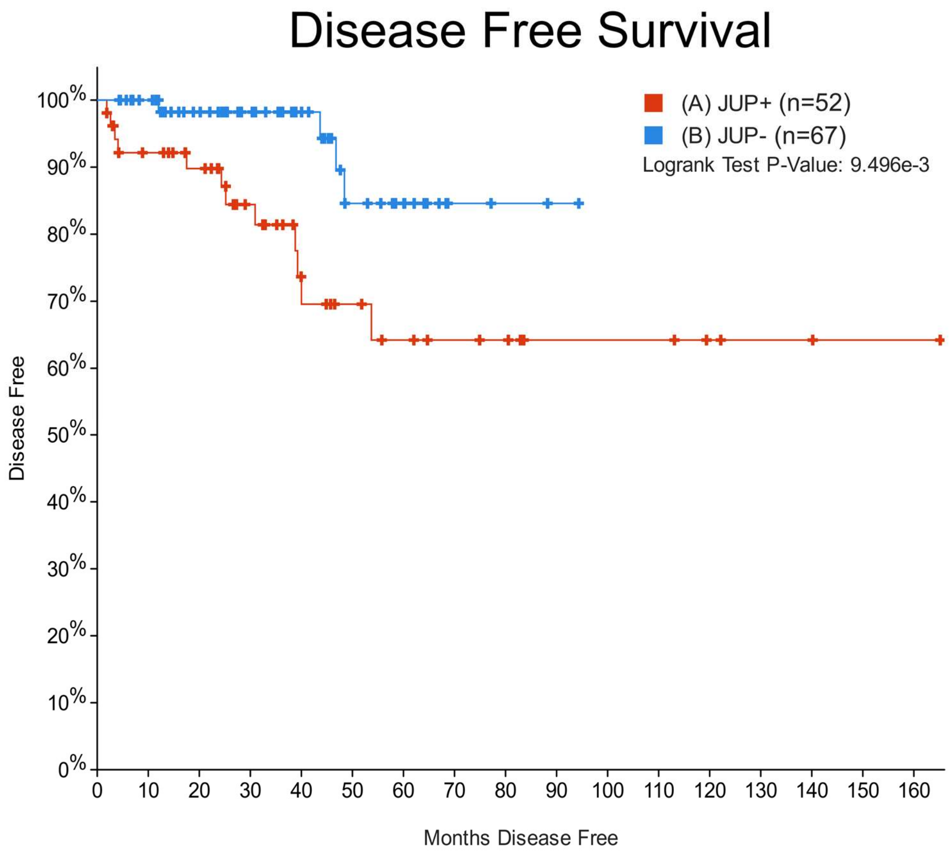

Figure S6: Combined analysis of JUP gene expression along with α-catenin (A) or β-catenin (B) gene expression for disease-free survival in the TCGA prostate cancer data set using cBioPortal. JUP, α-catenin, and β-Catenin mRNA expression z-scores were compared using a threshold ± 1 to define high and low expression, compared to expression in all diploid the tumor samples. Disease-free survival curves were calculated using the Kaplan-Meier method and Log-Rank test was applied to detect significant differences between groups (*p*-value as indicated in the graph).

(A)

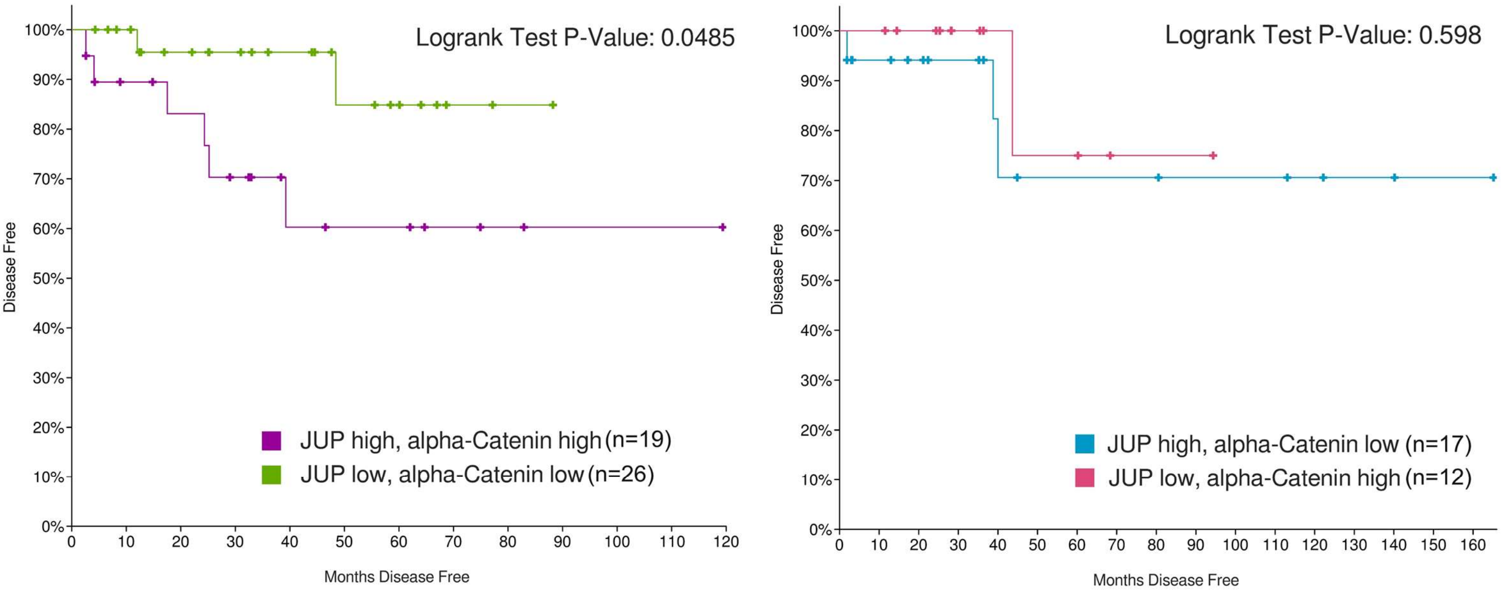

(B)

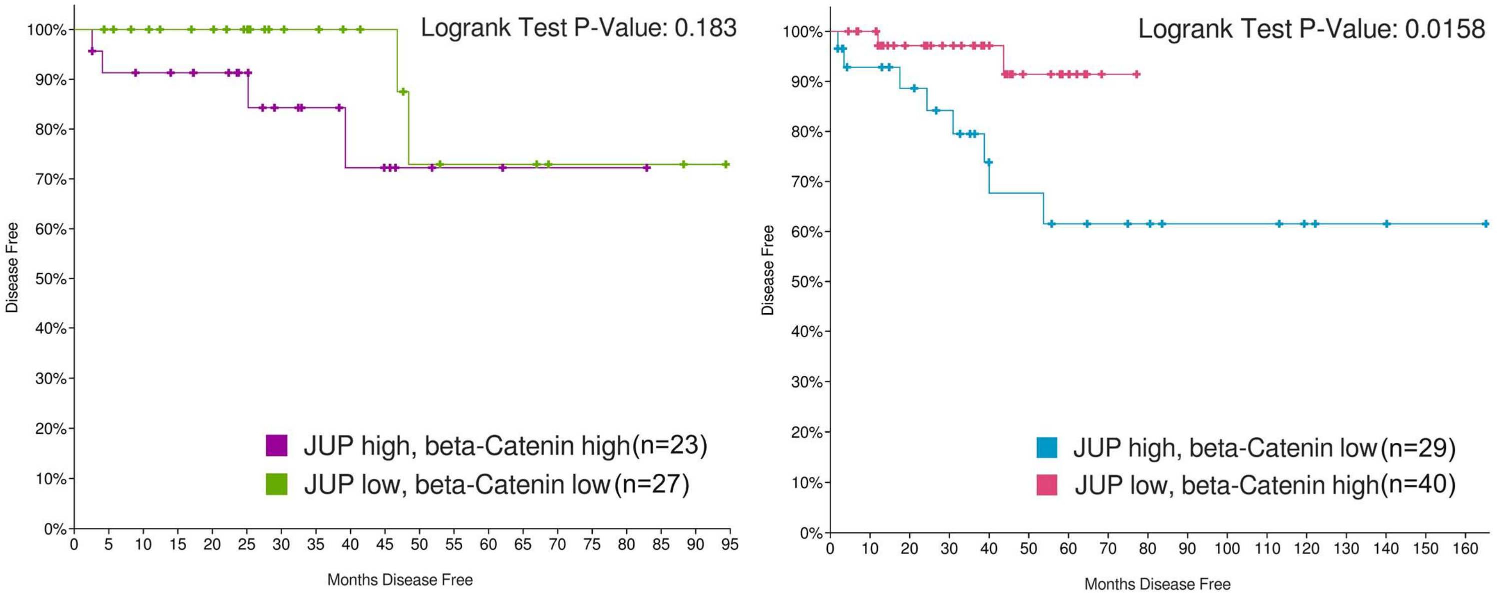

Figure S7: Genomic aberrations and JUP status. High JUP levels are associated with common genomic deletions (*MAP3K7, CHD1, PTEN, FOXP1*) in the ERG-negative subset, but not in the ERG-positive subset and are correlated with unfavorable outcome in the total cohort and the ERG-negative subset. Note that this association is reversed in the *CHD1*-deleted subset, where unfavorable outcome is associated with low JUP levels.

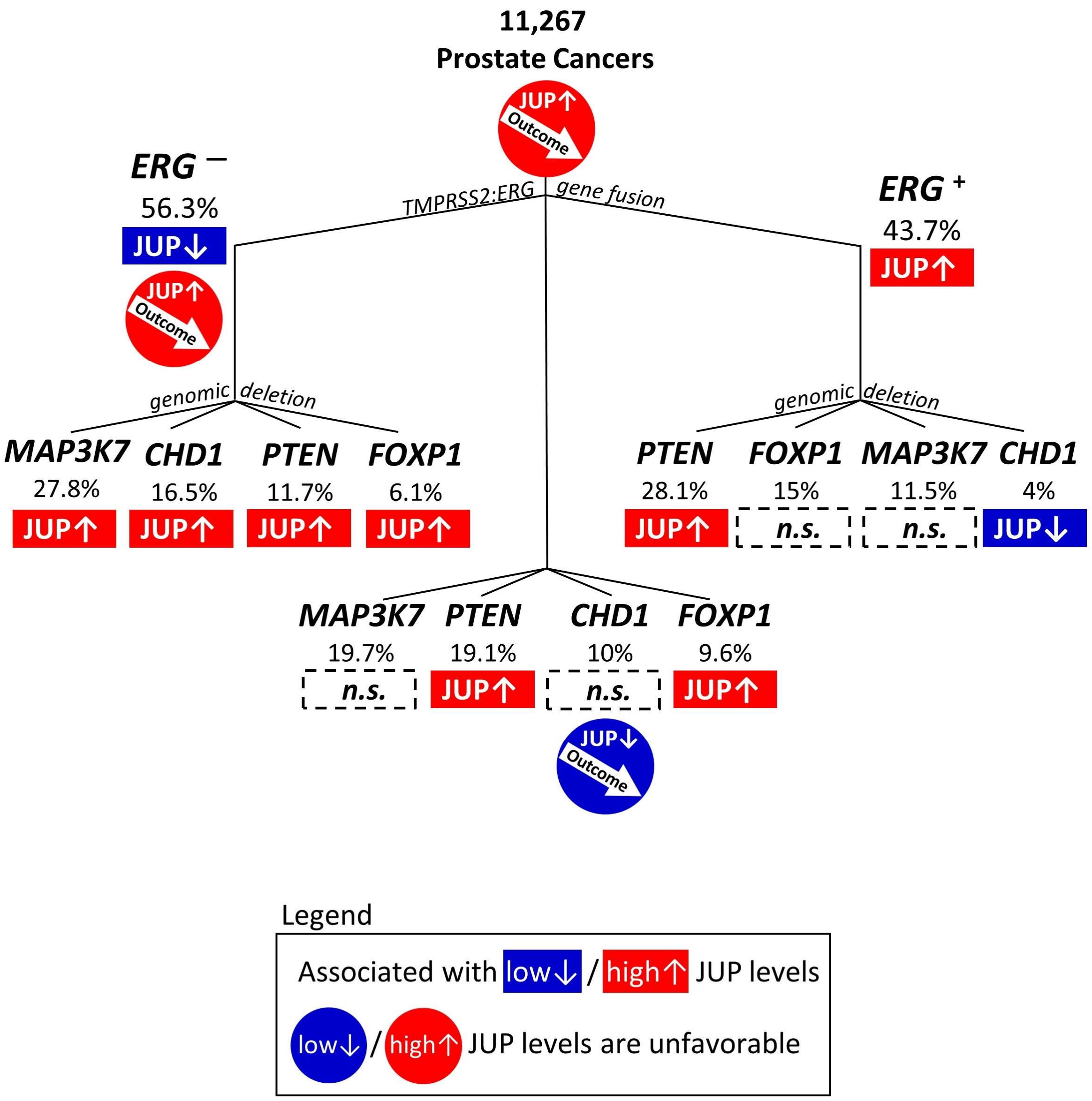

Supplement: Supplementary file 1 — Table S1. Association between ERG immunostaining results and PCa phenotype in all cancers. Table S2. Associations between trichotomized JUP immunostaining results and PCa phenotype in all cases analyzed. Table S3. Associations between trichotomized JUP immunostaining results and PCa phenotype in the TMPRSS2:ERG fusion‐negative subset. Table S4. Associations between trichotomized JUP immunostaining results and PCa phenotype in the TMPRSS2:ERG fusion‐positive subset. Table S5. Multivariate Cox regression analysis, including p‐values and hazard ratios for all comparisons. Fig. S1. Representative images of JUP immunostaining in cancerous prostate tissue. Fig. S2. Associations between JUP expression (trichotomized IHC staining tissue. Fig. S3. Correlation of JUP gene expression with ERG in PCa (TCGA data set), but not in benign/normal prostate gland (TCGA/GTEx combined data). Fig. S4. Influence of JUP on Wnt target gene expression in the TCGA prostate cancer data set analyzed using cBioPortal. Fig. S5. JUP gene expression and disease‐free survival in the TCGA prostate cancer data set analyzed using cBioPortal. Fig. S6. Combined analysis of JUP gene expression along with α‐catenin or β‐catenin gene expression for disease‐free survival in the TCGA prostate cancer data set using cBioPortal. Fig. S7. Genomic aberrations and JUP status (graphical abstract). [file MOL2-15-1956-s001.pdf]
